# Decoding the interplay of medical professionalism, mental well-being, and coping in undergraduate medical students across culture: using structural equation modeling

**DOI:** 10.3389/fmed.2024.1468654

**Published:** 2024-11-05

**Authors:** Kamran Sattar, Sultan Ayoub Meo, Muhamad Saiful Bahri Yusoff

**Affiliations:** ^1^Department of Medical Education, College of Medicine, King Saud University, Riyadh, Saudi Arabia; ^2^Department of Physiology, College of Medicine, King Saud University, Riyadh, Saudi Arabia; ^3^Department of Medical Education, School of Medical Sciences, University Sains Malaysia, Kota Bharu, Malaysia

**Keywords:** relationship, medical professionalism, mental well-being, coping, undergraduate, medical students, medical education, cultural contexts

## Abstract

**Introduction:**

The rigorous nature of medical education, long and night shifts, and prevalent issues like stress, anxiety, and depression affect medical students’ mental well-being and medical professionalism. This study aims to explore the intricate relationships between mental well-being, medical professionalism, and coping strategies, among undergraduate medical students, utilizing structural equation modeling (SEM) to unravel these dynamics.

**Methods:**

Conducted at Universiti Sains Malaysia, this cross-sectional study involved 234 medical students from the 1st, 3rd, and 5th years of the MBBS program. Data were collected via five validated survey instruments: DASS-9, TEQ, Dundee, Brief COPE, and CBI, through Google Forms. Participants were selected using purposive sampling. The surveys assessed mental well-being (burnout, anxiety, depression, stress), coping strategies, and medical professionalism attributes. Model fit was evaluated using established indices.

**Results:**

Findings indicated that professional behavior reduces burnout and negatively impacts negative coping strategies (NCSs). Additionally, medical professionalism indirectly enhances empathy and positively influences CSs. Conversely, psychological distress increases NCSs and reduces empathy. Positive coping strategies (PCSs) enhance empathy levels, while MWB issues elevate NCSs.

**Discussion:**

The study underscores the vital role of professional behavior in mitigating burnout and fostering positive coping mechanisms among medical students. Addressing MWB issues through targeted interventions can enhance empathy and professional behavior, ultimately improving the quality of patient care.

## Introduction

Medical students confront a myriad of stressors throughout their training, which inevitably takes a toll on their mental well-being. Comparisons with the overall populace reveal an increase in mental disorders among medical students, indicating a pressing concern for their holistic health (1). The demanding nature of medical education, characterized by the need to assimilate extensive information for examinations, adds an additional layer of pressure to an already challenging environment (2). Hence, the mental well-being of medical students is currently gaining significant attention globally, largely due to their essential role as the backbone of future healthcare systems (3, 4). It is clear that mental well-being issues, such as psychological distress (including stress, anxiety, and depression) and burnout, are prevalent among healthcare professionals (5, 6). Hence, unidentified and untreated burnout and a distressed life are sure to hurt their health (individual), their performance (interpersonal), and their level of care for patients (societal). It is still not fully understood how the above relationships work. Moreover, factors such as long work shifts (7), night shifts (8), and emerging issues like stress, anxiety, and depression (9) further exacerbate these challenges, casting a shadow over the well-being of medical students.

The goal of medical education is to develop doctors who are both healthy and competent so that they can better serve the community (10). However, in recent decades, the medical profession has faced heightened scrutiny and criticism for both perceived and actual violations of professional behavior. Numerous studies have been conducted on unprofessional conduct among trainee doctors. Research by Resnick et al. (59) found that professional misconduct is common among US general surgery residents and can lead to their dismissal from the training program. The challenges associated with unprofessional behavior do not arise solely after one qualifies as a physician. Research and medical authorities indicate that the issues often stem from the early years of medical training. The General Medical Council underscores the importance of fostering professional behaviors in medical students starting from their initial undergraduate years (60). There are growing concerns about the decline in professionalism among students (61) and its links to reduced job performance and burnout (62). The significance of medical professionalism is gaining renewed attention among healthcare professionals and society to address these concerns and enhance awareness of the social responsibilities of medicine. Additionally, it has become increasingly recognized and accepted in recent years that the most effective approach is to realign medical education and practice with its fundamental values of professionalism (63). It is, therefore, essential to enhance students’ learning around the attributes of medical professionalism during undergraduate medical education (64). However, a concerning trend of declining professionalism attributes among medical students during their training poses a significant threat to professionalism and, consequently, patient care quality (11). The scope of medical professionalism differs throughout the world with different cultural contexts (12) but typically includes values such as altruism, accountability, and a commitment to excellence, as recognized by organizations like the American Board of Internal Medicine (13). Recognizing the pivotal role that medical students play in shaping the future of healthcare systems, it becomes imperative to address issues related to mental well-being, including burnout and psychological distress, to ensure the cultivation of empathetic and ethical healthcare professionals (3, 4).

Coping refers to the mental, emotional, and behavioral efforts made to manage a challenging relationship between an individual and their environment (14). Research has shown that both individual characteristics and situational factors can affect coping. In a review, Skinner et al. (15) noted that while hundreds of coping strategies are currently under evaluation, these strategies generally fall into five primary categories: problem-solving, support-seeking, avoidance, diversion, and positive cognitive restructuring.

At the undergraduate level, utilisation of coping strategies (CSs) for discrete mindfulness and the level of medical students’ mental well-being might play a crucial role in leading them towards medical professionalism attributes or even an unprofessional act (i.e., professionalism laps). There is a global trend in the use of both positive and negative coping strategies (NCSs). Problem-focused coping seeks to reduce or eliminate the source of stress, while emotion-focused coping changes the way an individual responds to stressors.

Literature suggests that CSs are vital for well-being (16). According to Zajacova and Lynch (17), stress is prevalent among students in academic settings. Therefore, a student’s ability to choose and employ effective coping strategies (CS) to mitigate psychological distress is linked to their other personal resources (18). Such situations highlight the necessity of developing a plan to identify the relationships between medical professionalism and mental well-being. This approach can assist medical educators in recognizing, exploring, and improving their strategies to address instances of unprofessional behavior among students (19).

Identifying these interrelations will allow medical educators to positively influence students’ professional behavior, enhancing their understanding of professionalism. This, in turn, will help achieve the fundamental goal of medical education: producing “well-being doctors” capable of addressing the needs of patients and the community.

**Figure 1:**
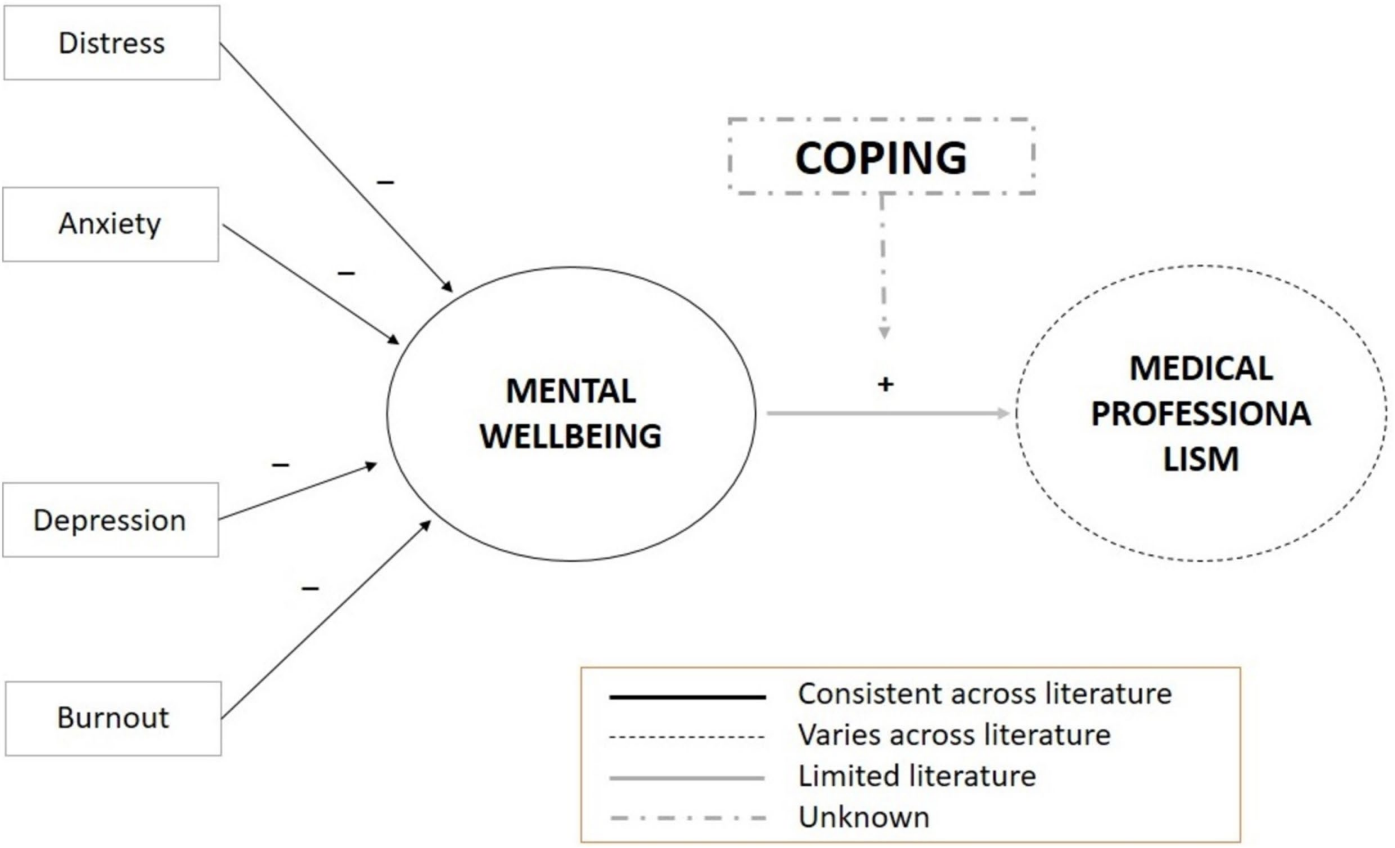
Theoretical framework.

As depicted in Figure 1, the existing literature reveals a scarcity of evidence regarding the impact of mental well-being issues on the professionalism of medical students. Current reports fail to adequately explore the causal relationships between these two critical domains: mental well-being and medical professionalism. Additionally, there is a notable gap in our understanding of the role of coping mechanisms as a mediator in the relationship between mental well-being indicators (including stress, anxiety, depression, and burnout) and attributes of medical professionalism (such as professional behavior and empathy).

Despite the paramount importance of understanding the intricate relationship between coping strategies (CSs), mental well-being, and medical professionalism among medical students, this area remains relatively unexplored in the existing literature. Exploring these interconnections holds significant promise for unravelling the complex dynamics that influence individual health, interpersonal relationships, and ultimately, the quality of patient care (5, 6). By investigating these relationships, researchers can shed light on how the structure and content of medical education programs impact student well-being and medical professionalism, paving the way for targeted interventions and improvements in medical training practices.

The objective of our study is to examine the causal-effect relationship of medical professionalism, CSs, and mental well-being issues using structural equation modeling. Therefore, the relevant research question we have; what is the relationship amid medical professionalism, mental well-being and coping strategies among healthcare professionals?

We have formulated the following hypotheses aligning with our objective to examine the causal-effect relationships using structural equation modeling (SEM). These shall guide the analysis of the relationships between medical professionalism, coping strategies, and mental well-being.

*Hypothesis 1 (H1)*. Higher levels of medical professionalism are associated with better mental well-being among healthcare professionals.

*Hypothesis 2 (H2)*. Mental wellbeing issues negatively influence medical professionalism.

*Hypothesis 3 (H3)*. Mental well-being issues hinder usage of positive coping.

*Hypothesis 4 (H4)*. Mental well-being issues are associated with negative coping.

## Methodology

### Study design

Authors selected quantitative correlational study design for this study as it allows for the rigorous examination of causal relationships and complex interactions among variables, providing valuable insights that can inform practice and policy.

### Setting

The medical curriculum at Universiti Sains Malaysia follows the student-centered, problem-based learning, integrated teaching, community-based, electives and systematic (SPICES) educational model, spanning 5 years with pre-clinical and clinical phases. The pre-clinical years focus on fundamental biology and early clinical exposure, while the clinical phase emphasizes practical skills in real healthcare settings. This study was conducted using Google Forms, offering accessibility, cost-effectiveness, time efficiency, and scalability in data collection, aligning with modern research practices.

### Participants

Data were collected using five survey questionnaires from 234 subjects. Kline (65) suggests a minimum sample size of 200 subjects for structural equation modeling (SEM) analysis. Factoring in a 30% non-response rate, the estimated sample size required for this study was 323 medical students.

At the beginning, we distributed the questionnaires to all undergraduate medical students studying in the years 1, 3 and 5 at Universiti Sains Malaysia, Health Campus, Kelantan. After the formal announcements (through student class leaders), the online link for the survey was sent to only those who showed their willingness by replying through emails or WhatsApp messages. They were from diverse levels of study (years 1, 3, and 5). The class leaders’ announcements for the research were made so that the interested student may contact the concerned person who shall discuss with students all the details about the research surveys (e.g., consent, time, date, venue, duration, potential benefits, and potential risks).

### Data collection

The primary data for structural equation modeling (SEM) came from responses gathered via five survey questionnaires in a cross-sectional study. A purposive sampling method was applied to 234 medical students across the 1st, 3rd, and 5th years at Universiti Sains Malaysia’s Health Campus in Kelantan. The purpose of collecting data from medical students in their first, third, and fifth years was to expect a variety of responses and to learn about and collect a variety of factual situations and life experiences experienced by medical students at different phases about CSs, mental well-being issues (MWBi), and medical professionalism, throughout their undergraduate medical career. During the cross-sectional study, we investigated the level of mental well-being (burnout, anxiety, depression, stress) CSs, and medical professionalism attributes at a single time frame (October 2022–November 2022) using validated instruments listed with their respective determinants in Table 1.

**Table 1:** Five survey questionnaires with respective determinants.

Questionnaire	Determinants
Dundee Polyprofessionalism Inventory I: Academic Integrity (Dundee Inventory)	Professional behavior score
Depression, Anxiety, and Stress Scales DASS-9	Depression, anxiety, & stress score
Brief COPE	Coping score
Copenhagen Burnout Inventory	Burnout score
Toronto Empathy Questionnaire	Empathy score

All the survey questionnaires are included in Appendix N. DASS-9, Depression, Anxiety and Stress Scale; Brief COPE, Brief Coping Orientation to Problems Experienced; CBI, Copenhagen Burnout Inventory; TEQ, Toronto Empathy Questionnaire.

A detailed meeting among the research team was held to finalize the inventories, a consensus was achieved on the list of instruments, and later in a subsequent meeting, an agreement was sought on selecting items from the selected inventories to be utilized. The criteria for selecting or omitting items from the selected inventories were based on our objectives. Two inventories, i.e., Distress Anxiety Stress Scale-9 (DASS-9) and Toronto Empathy Questionnaire (TEQ) were selected to be used as it is, whereas three inventories, i.e., Dundee, Brief Coping Orientation to Problems Experienced (COPE), and Copenhagen Burnout Inventory (CBI) were used with reduced items to best match our objectives. For the Dundee inventory, we selected eight items (from a total of 34), Brief COPE; 12 items (from a total of 28), and CBI; 13 items (from a total of 19).

As mentioned earlier, to best depict the construct of interest, from Dundee inventory, we selected and utilized eight types of professionalism lapse items. The Dundee inventory (Poly-professionalism Inventory I: Academic Integrity), developed and validated by Roff et al. (20, 21), has been frequently used as customized (22, 23). The author of the brief COPE inventory, Charles S. Carver has allowed to use this inventory as complete or any scales relevant to the researchers’ needs. Brambila-Tapia et al. (24) used reduced items (14) for their study. We also performed an item reduction for Brief COPE, leaving only the most representative subscales for positive (adaptive) and negative (maladaptive) coping. This way, we were able to have 6 items relevant to each (positive and negative) coping strategy. Concerning CBI, out of the 19, we identified and used 13 survey items to best represent the constructs of interest. It’s a practice of researchers to carefully select which type of burnout among the three (personal, work, and client-related) to be selected, as Huang et al. (25) did not measure work-related burnout but only personal-related and patient-related. Considering our research needs, we also did not measure client-related burnout as it did not match our research objectives.

A summary detailing the item distribution, with response categories and score interpretation, is available (Table 2). Five inventories with the final items are mentioned in Appendix N. Furthermore, we evaluated model fit using established indices (Table 3) supported by widespread literature.

**Table 2:** Detailed summary of validated study instruments.

Instrument	Domains	No. of items	Response categories	Score interpretation
Dundee Inventory	Unprofessional behavior	8	“1. Ignore (None)” “2. Reprimand (verbal warning)” “3. Reprimand (written warning)” “4. Reprimand, plus mandatory counseling” “5. Reprimand, counseling, extra work assignment” “6. Failure of specific class/remedial work to gain credit” “7. Failure of a specific year (repetition allowed)” “8. Expulsion from college (readmission after one year possible)” “9. Expulsion from college (no chance for readmission)” “10. Report to a regulatory body”	Mean and SD
Depression, Anxiety and Stress Scale (DASS-9)	Depression	3	“0—Did not apply to me at all” “1—Applied to me to some degree, or some of the time” “2—Applied to me to a considerable degree or a good part of the time” “3—Applied to me very much, or most of the time”	Normal scores were treated as negative screening, and mild to extremely severe were treated as positive screening
	Anxiety	3		
	Stress	3		
Brief Coping Orientation to Problem Experienced (Brief COPE)	Denial	2	“1—I have not been doing this at all” “2—I have been doing this a little bit” “3—I have been doing this a medium amount” “4—I have been doing this a lot”	Mean and SD
	Substance use	2		
	Support-emotional	2		
	Self-blame	2		
	Religion	2		
	Acceptance	2		
Copenhagen Burnout Inventory (CBI)	Personal-related	6	“100—Always/To a very high degree” “75—Often/To a high degree” “50—Sometimes/Somewhat” “25—Seldom/To a low degree” “0—Never/Almost never/To a very low degree”	Average scores were calculated for each domain An average score of 50 or above is treated as burnout
	Work-related	7		
Toronto Empathy Questionnaire (TEQ)	Empathy-positive worded items	8	“Never = 0” “Rarely = 1; Sometimes = 2” “Often = 3” “Always = 4”	Sores are summed to derive the total for the Toronto Empathy Questionnaire, which can range from 0 to 64
	Empathy-negative worded items	8		

**Table 3:** Various fit indices with levels of acceptance.

Name of category	Name of index	Level of acceptance
Absolute fit	“Root mean square for error approximation” (RMSEA)	<0.08 (69)
	“Goodness of fit index” (GFI)	>0.9 (70)
Incremental fit	“Comparative fit index” (CFI)	>0.9 (71)
	“Tucker–Lewis index” (TLI)	>0.9 (72)
	“Normed fit index” (NFI)	>0.9 (73)
Parsimonious fit	“Chi-square/degree of freedom” (v2/df)	<5 (74)

Since, the SEM utilized the maximum likelihood (ML) method, known for its asymptotically unbiased and efficient estimator (26), it helped us prioritize minimizing bias in the analysis process to ensure accurate results.

### Statistical method

Using SPSS version 20, we conducted a descriptive analysis of demographic information. Samples having full responses to all inventories underwent SEM analysis using Analysis of Moment Structure (AMOS 29). We utilized five validated scales to meet research needs and conducted confirmatory factor analysis (CFA) to test measurement model fit for each construct.

### Measurement model

CFA delineated observed variables for latent constructs and model fit was assessed for all items across five inventories. Composite reliability (CR) was calculated using Microsoft^®^ Excel^®^ with the following formula;


$$\frac{{\left(\sum \lambda_{i}\right)}^{2}}{{\left(\sum \lambda_{i}\right)}^{2}+\sum \varepsilon_{i}}$$

whereby *λ* (lambda) is the standardized factor loading for item *i* and *ε* is the respective error variance for item *i*. The error variance *ε* is estimated based on the value of the standardized loading (*λ*) as *ε_i_* = 1 − *λ*^2^*_i_*.

### Steps of SEM analysis

The researcher prioritized minimizing bias in the analysis process to ensure accurate results. SEM utilized the maximum likelihood (ML) method, known for its asymptotically unbiased and efficient estimator (26). Moreover, we followed five logical steps in SEM: (1) model specification, (2) identification, (3) parameter estimation, (4) model evaluation, and (5) model modification (66–68). Our study aimed to identify causal relationships between medical professionalism and mental well-being, ensuring association, time order, and non-spuriousness. Association between variables, indicating correlation, was a starting point for establishing causality, emphasizing statistical significance. Time order was crucial; the cause must precede the effect logically. Non-spurious relationships were sought through study design and meticulous data collection. In SEM, path analysis diagrams were created, and analyses were conducted following AMOS guidelines. Standardized estimates were utilized for their consistency across variables with different measurement scales. This approach allowed the comparison of exogenous variables’ (with no arrows pointing to, are exogenous variables) effects on endogenous variables (the variables to which arrows point are endogenous), facilitating the recognition of causal relationships.

## Results

### Demographic data

Survey questionnaires were distributed to a total of 800 students and 234 students participated, including 95 (40.6%) from the first, 41 (17.55) third, and 98 (41.9) fifth year of the medical course. Participants were primarily female (78.2%), the overriding race was Malaysian (76.9%), and the majority (41.9%) were from the fifth year of study. The demographic distribution is highlighted in Table 4.

**Table 4:** Demographics of the study participants (*n* = 234) for surveys.

Demographic characteristics (*n* = 234)	*n* (%)
Medical course	
1^st^ year	95 (40.6)
3^rd^ year	41 (17.5)
5^th^ year	98 (41.9)
Personal demographics	
Gender	
Male	49 (20.9)
Female	183 (78.2)
Prefer not to say	2 (0.9)
Race	
Malay	180 (76.9)
Indian	26 (11.1)
Chinese	22 (9.4)
Other	6 (2.5)

### Measurement and structural model findings

Table 5 shows the CFA findings and helped to assess model fit with measurement models of professional behavior (Appendix B) and mental well-being (Appendix C). Similarly, Appendix D depicts a model for positive coping strategies (PCS) which was evaluated using six items. However, initial attempts to model negative coping strategies (NCS1) were unsuccessful due to poor factor loading of two items (Appendix E), which were subsequently removed. The revised model (NCS2) with four items (Appendix F) demonstrated acceptable fit indices (Table 3). Moreover, the burnout model (Appendix G) comprised 13 items and achieved a satisfactory model fit. Likewise, empathy (Appendix H) with 16 items attained model fit.

**Table 5:** Results of CFA for all inventories (the measurement model of constructs).

Inventories		Determinants	CFA parameters obtained					
			*χ*^2^/df=	*p*	TLI	CFI	RMSEA	CR
Polyprofessionalism		Professional behavior	1.005	0.441	1.000	1.000	0.005	0.765
DASS-9		Depression, anxiety, and stress	1.127	0.319	0.992	0.996	0.023	0.830
Brief COPE	^*^NCS2	Negative coping strategies (4 items)	3.681	0.055	0.948	0.991	0.107	0.596
	^**^PCS	Positive coping strategies (6 items)	1.352	0.221	0.991	0.996	0.039	
CBI		Burnout	1.020	0.434	0.999	1.000	0.009	0.928
TEQ		Empathy	0.820	0.855	1.000	1.000	<0.001	0.138

Depression, Anxiety and Stress Scale (DASS-9), ^*^Negative Coping Strategies (NCS), ^**^Positive Coping Strategies (PCS); taken from Brief Coping Orientation to Problems Experienced (Brief COPE), Copenhagen Burnout Inventory (CBI), Toronto Empathy Questionnaire (TEQ). Chi-square (*χ*^2^), Degree of freedom (df), *p*-value (*p*), Tucker–Lewis index (TLI), Comparative fit index (CFI), Root mean square error of approximation (RMSEA), Composite reliability (CR).

Appendix I depicts the results of each latent construct with relevant manifest constructs.

During SEM, the Variable terms used for our structural models with respective intended concepts are enlisted in Table 6. Results of the goodness of fit indices for the mental well-being (MWB) and medical professionalism (MP) relationship, with mediating effects of CSs (positive and negative), are summarized in Table 7.

**Table 6:** Variables terms used for our SEM models with respective intended concepts.

Variable terms	Intended concepts
PDScore	Psychological distress
BurnoutScore	Burnout
TEQScore	Empathy
PolyScore	Professional behavior
PCS	Positive coping strategy
NCS	Negative coping strategy

**Table 7:** The goodness of fit indices for supporting the best-fit model.

Models	The goodness of fit indices						
	*χ*^2^/df	*p*-value	TLI	CFI	RMSEA	GFI	NFI
Model 1 (MWBi-MP)	10.549	0.001	0.608	0.935	0.202	0.979	0.931
Model 2 (modified MWBi-MP)	1.459	0.227	0.981	0.997	0.044	0.997	0.990
Model 3 (MWBI-PCS-MP)	1.456	0.045	0.973	0.982	0.044	0.965	0.947
Model 4 (MWBi-NCS-MP)	2.193	0.000	0.946	0.966	0.072	0.949	0.941

Chi-square (*χ*^2^), Degree of freedom (df), *p*-value (*p*), Tucker–Lewis index (TLI), Comparative fit index (CFI), Root mean square error of approximation (RMSEA), Goodness of fit index (GFI), Normed fit index (NFI).

**Figure 2:**
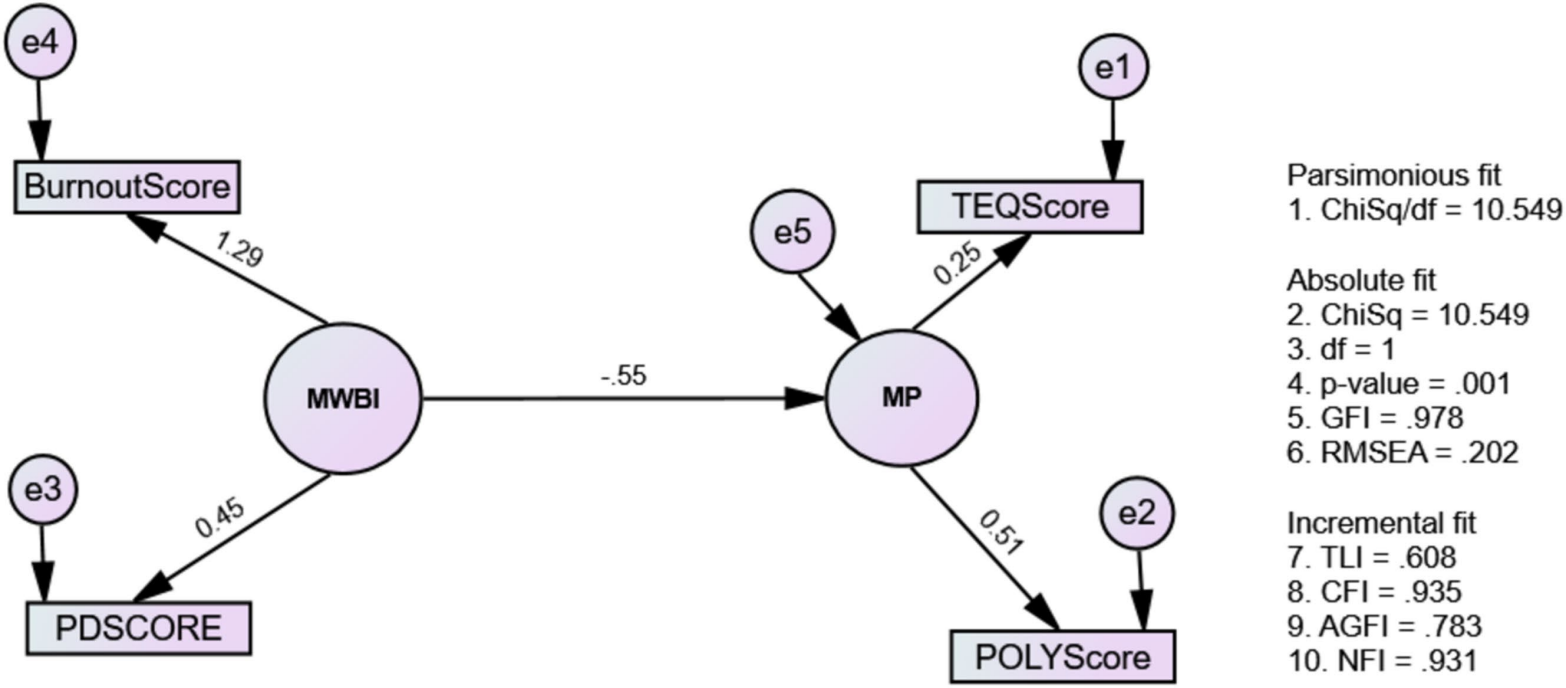
The causal-effect relationship between mental well-being issues and medical professionalism (Model 1: MWBi-MP).

Our study revealed that initial Model 1 (Figure 2), examining causal-effect relationships, did not fit well despite good GFI (0.979) and acceptable CFI (0.935). Model modification improved fit to create Model 2.

**Figure 3:**
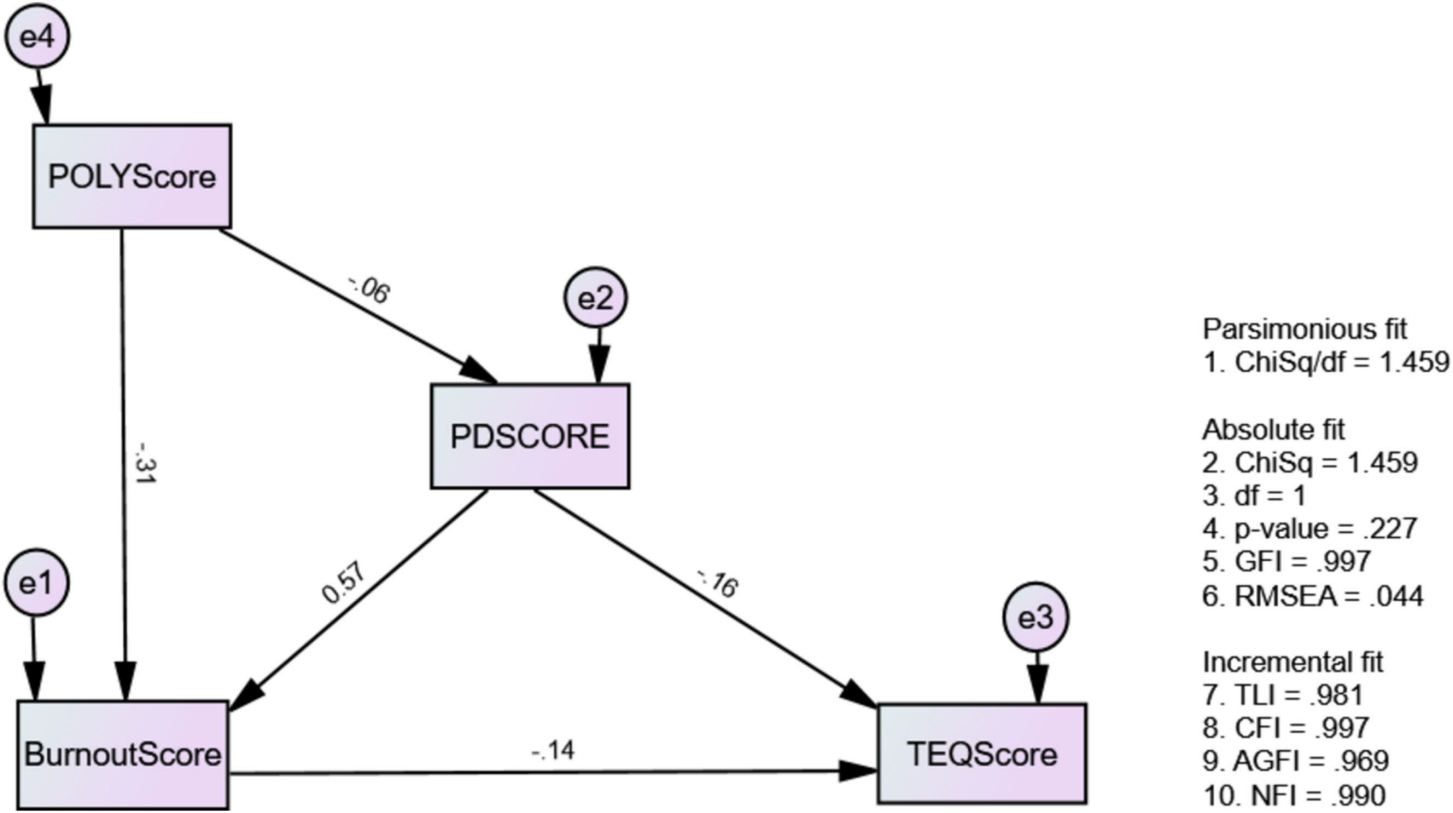
The modified causal-effect relationship between mental well-being issues and medical professionalism (Model 2: modified MWBi-MP).

Figure 3 shows Model 2 which exhibited better-fit indices: Chi-square/df = 1.459, TLI = 0.981, CFI = 0.997, RMSEA = 0.044, GFI = 0.997, NFI = 0.990 (Table 7). No post-hoc modifications were needed due to the good fit index. Furthermore, Model 2’s direct, indirect, and total effects on the model paths are presented in Tables 8, 9.

**Table 8:** The estimates of standardized and unstandardized regression weights of variables for Model 2.

Observed variables		*β*	*B*	SE	*p*-values
Professional behavior (PolyScore)	Psychological Distress (PDScore)	−0.059	−0.025	0.028	0.371
	Burnout (BurnoutScore)	−0.306	−0.268	0.043	<0.001
Psychological distress (PDScore)	Burnout (BurnoutScore)	0.567	1.174	0.102	<0.001
	Empathy (TEQScore)	−0.159	−0.254	0.125	0.042
Burnout (BurnoutScore)	Empathy (TEQScore)	−0.136	−0.105	0.060	0.082

*β = standardized regression weights; B = unstandardized regression weights; SE = standard error.*

**Table 9:** The unstandardized and standardized estimates of direct, indirect, and total effects for variables relationships for Model 2.

Parameter	Variables relationship		Total (95% CI)	Direct (95% CI)	Indirect (95% CI)
Unstandardized	PolyScore	PDScore	−0.025 (−0.078, 0.034)^***^	−0.025 (−0.078, 0.034)^***^	—
	PolyScore	BurnoutScore	−0.297 (−0.407, −0.176)^**^	−0.268 (−0.358, −0.176)^**^	−0.029 (−0.089, 0.040)^***^
	PDScore		1.174 (0.950, 1.384)^**^	1.174 (0.950, 1.384)^**^	—
	PolyScore	TEQScore	0.037 (0.001, 0.092)^*^	—	0.037 (0.001, 0.092)^*^
	PDScore		−0.377 (−0.578, −0.177)^**^	−0.254 (−0.507, 0.013)^***^	−0.123 (−0.306. 0.037)^***^
	BurnoutScore		−0.105 (−0.248, 0.032)^***^	−0.105 (−0.248, 0.032)^***^	—
Standardized	PolyScore	PDScore	−0.059 (−0.186, 0.076)^***^	−0.059 (−0.186, 0.076)^***^	—
	PolyScore	BurnoutScore	−0.340 (−0.466, −0.200)^**^	−0.306 (−0.404, −0.190)^**^	−0.033 (−0.101, 0.048)^***^
	PDScore		0.567 (0.474, −0.649)^**^	0.567 (0.474, 0.649)^**^	—
	PolyScore	TEQScore	0.055 (0.001, 0.130)^*^	—	0.055 (0.001, 0.130)^*^
	PDScore		−0.236 (−0.358, −0.109)^**^	−0.159 (−0.313, 0.010)^***^	−0.077 (−0.181, 0.024)^***^
	BurnoutScore		−0.136 (−0.309, 0.049)^***^	−0.136 (−0.309, 0.049)^***^	—

Bootstrap (1000) with 95% bias-corrected confidence interval: L = lower bound; U = upper bound; ^*^p-value <0.05; ^**^p-value <0.01; ^***^p-value >0.05.

Our study revealed that psychological distress significantly reduced empathy levels in undergraduate medical students. Additionally, psychological distress significantly contributed to burnout (*β* = 0.567, *p*-value <0.001) in undergraduate medical students (Table 8). This was in align with our predicted H2. Hypothesis (H1) predicted, and our study also revealed that there was a significant influence of professional behavior on burnout (*β* = −0.306, *p* < 0.001). Additionally, professional behavior significantly affects empathy indirectly (*β* = 0.055) as shown in Table 9.

**Figure 4:**
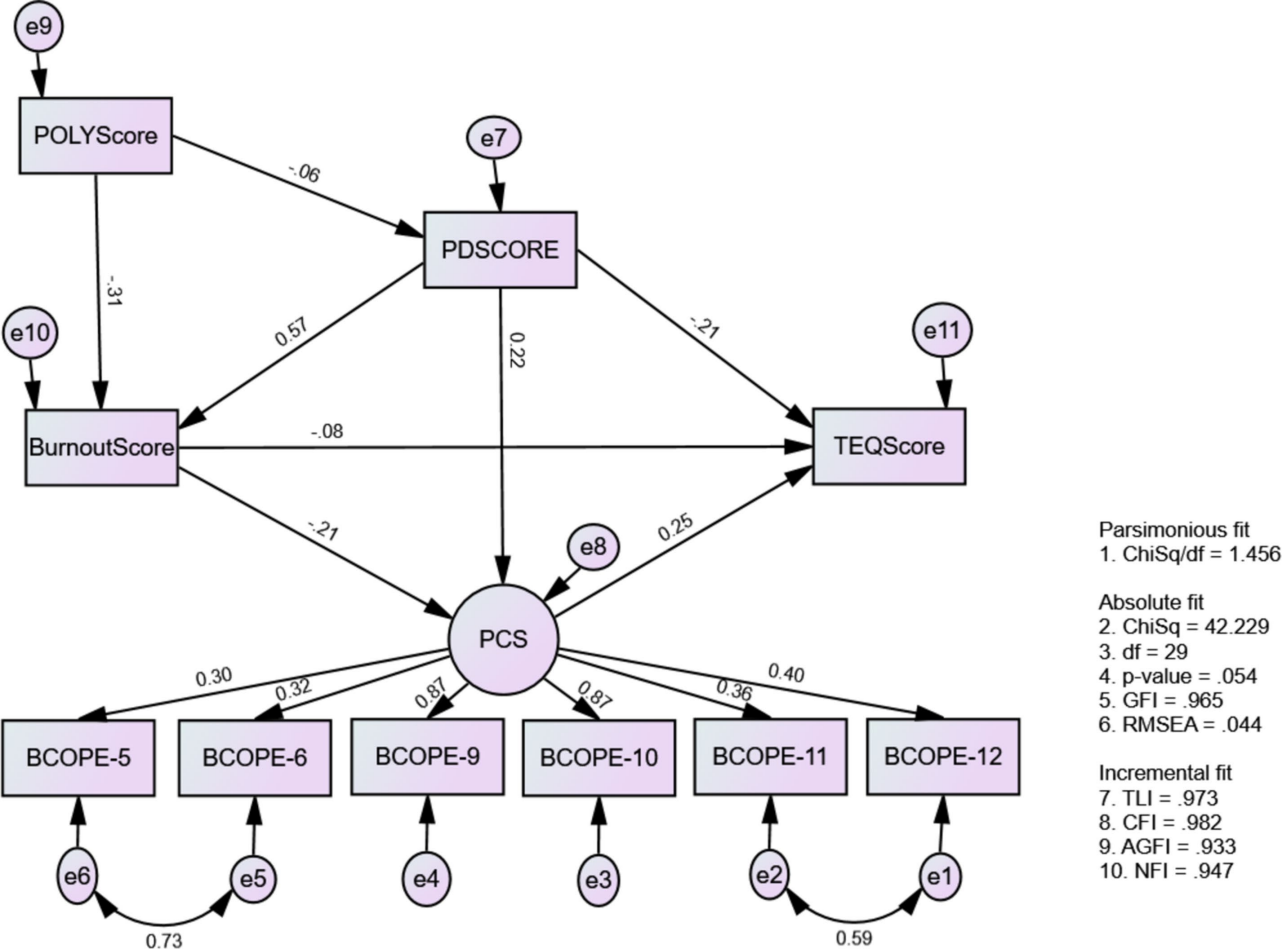
The causal-effect relationship model among mental well-being issues, positive coping strategy, and medical professionalism (Model 3: MWBi-PCS-MP).

According to our results, Model 3 achieved acceptable fit indices (Table 7) and underwent modifications based on AMOS recommendations to enhance theoretical coherence. Specifically, adjustments included covariances between indicators (BCOPE5 and BCOPE6) and (BCOPE11 and BCOPE12) for the latent variable PCS (Figure 4). The results of this model informed that psychological distress influenced the use of positive coping strategies. As predicted by H3, psychological distress acted as an inhibitor for positive coping strategies, mediating through burnout and significantly reducing their use (*p* < 0.05). This model also confirmed that positive coping strategies as contributing factors for empathy.

Results also indicate that students’ ability to employ positive coping strategies decreases with burnout. Despite psychological distress prompting the use of positive coping strategies, its mediating effect through burnout significantly reduces their utilization among medical students. Furthermore, positive coping strategies enhance empathy, with a standardized direct effect of 0.253 (Tables 10, 11) this was predicted in H4.

**Table 10:** The estimates of standardized and unstandardized regression weights of variables for Model 3.

Endogenous variables		*β*	*B*	SE	*p*-values
Burnout (BurnoutScore)	Empathy (TEQScore)	−0.083	−0.064	0.059	0.281
	Positve coping strategy (PCS)	−0.207	−0.007	0.003	0.024
Psychological distress (PDScore)	Empathy (TEQScore)	−0.214	−0.341	0.123	0.006
	Positive coping strategy (PCS)	0.216	0.016	0.007	0.019
Positive coping strategy (PCS)	Empathy (TEQScore)	0.253	5.476	1.706	0.001

*β = standardized regression weights; B = unstandardized regression weights; SE = standard error. Estimates for PDScore → BurnoutScore, PolyScore → PDScore, PolyScore → BurnoutScore remain the same as in Table 9, hence not included here.*

**Table 11:** The unstandardized and standardized estimates of direct, indirect, and total effects for variables relationships for Model 3.

Parameter	Variables relationship		Total (95% CI)	Direct (95% CI)	Indirect (95% CI)
Unstandardized	PolyScore	PDScore	−0.025 (−0.078, 0.034)^***^	−0.025 (−0.078, 0.034)^***^	—
	PolyScore	PCS	0.002 (0.000, 0.005)^*^	—	0.002 (0.000, 0.005)^*^
	PDScore		0.007 (−0.004, 0.019)^***^	0.016 (0.005, 0.033)^**^	−0.009 (−0.020–0.001)^*^
	BurnoutScore		−0.007 (−0.017, −0.001)^*^	−0.007 (−0.017, −0.001)^*^	—
	BurnoutScore	TEQScore	−0.105 (−0.248, 0.032)^***^	−0.064 (−0.187, 0.073)^***^	−0.041 (−0.101, −0.007)^*^
	PCS		5.476 (1.992, 10.451)^**^	5.476 (1.992, 10.451)^**^	—
Standardized	PolyScore	PDScore	−0.059 (−0.186, 0.076)^***^	−0.059 (−0.186, 0.076)^***^	—
	PolyScore	PCS	0.058 (−0.004, 0.129)^***^	—	0.058 (−0.004, 0.129)^***^
	PDScore		0.098 (−0.057, 0.236)^***^	0.216 (0.048, 0.385)^**^	−0.118 (−0.238, −0.012)^*^
	BurnoutScore		−0.207 (−0.399, −0.014)^*^	−0.207 (−0.399, −0.014)^*^	—
	BurnoutScore	TEQScore	−0.136 (−0.309, 0.049)^***^	−0.083 (−0.253, 0.098)^***^	−0.053 (−0.129, −0.008)^*^
	PCS		0.253 (0.102, 0.388)^**^	0.253 (0.102, 0.388)^**^	—

Bootstrap (1000) with 95% bias-corrected confidence interval: L = lower bound; U = upper bound; ^*^p-value <0.05; ^**^p-value <0.01; ^***^p-value >0.05. Estimates for PolyScore → BurnoutScore, PDScore → BurnoutScore, PolyScore → TEQScore, PDScore → TEQScore remain similar as in Table 10, hence not included here.

**Figure 5:**
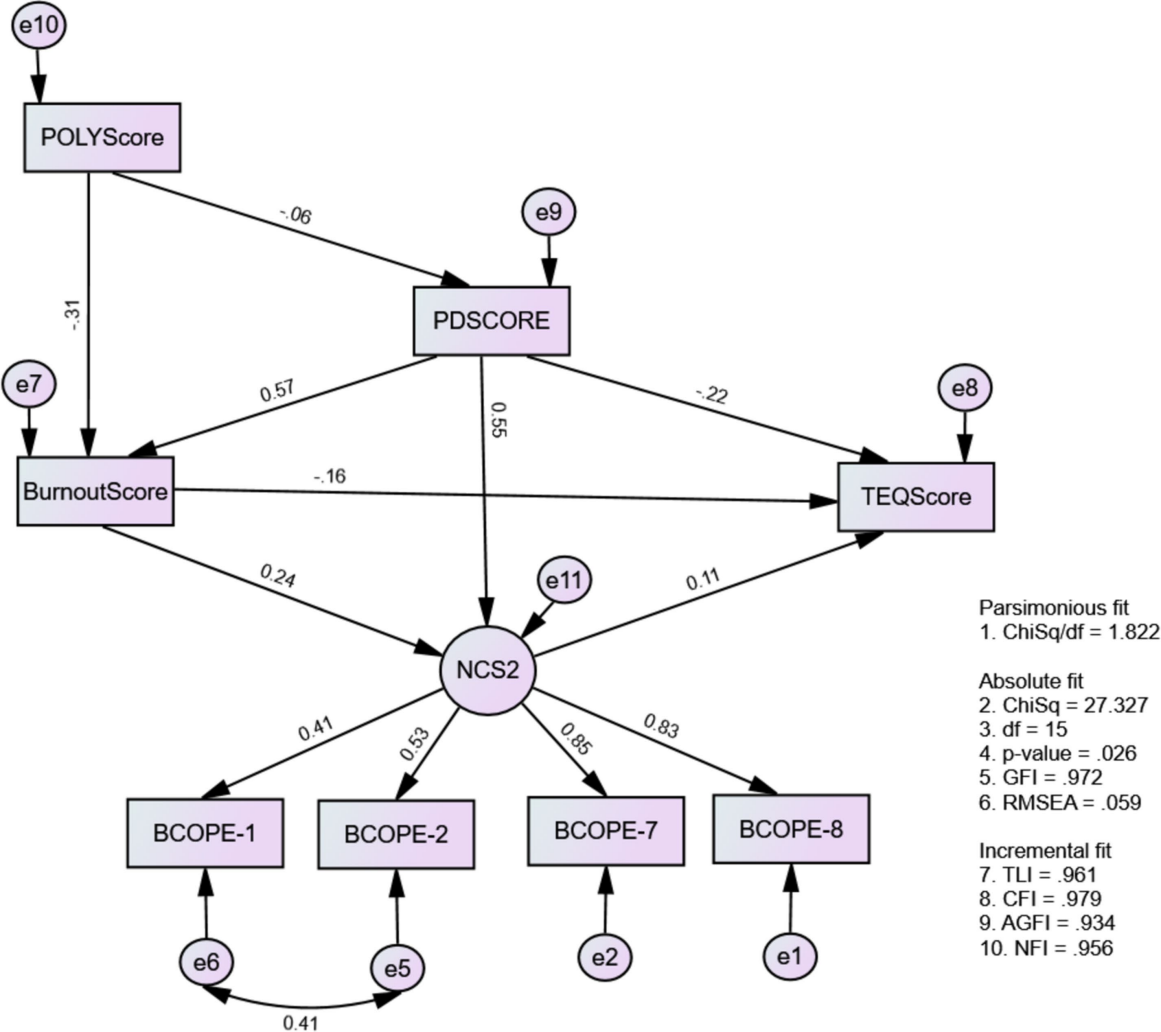
The causal-effect relationship model among mental wellbeing issues, negative coping strategy, and medical professionalism (Model 4: MWBi-NCS2-MP).

This study further revealed that Model 4 (Figure 5) helped us investigate the effects of NCS2 on medical students’ medical professionalism and mental well-being. This model has met the criteria for model fit: Chi-square/df = 1.822, *p* = 0.026, TLI = 0.946, CFI = 0.961, RMSEA = 0.059, GFI = 0.972, and NFI = 0.956 (Table 7). Moreover, as predicted by H4, the results regarding negative coping strategies (NCS), indicated that mental well-being issues (burnout and psychological distress) significantly contributed to NCS. While professional behavior does not directly influence NCS2, a significant indirect effect was observed as an inhibitor through burnout (*β* = −0.113, *p* < 0.05) for negative coping strategies. This implied that professional behavior decreases the usage of NCS among medical students (Tables 12, 13).

**Table 12:** The estimates of standardized and unstandardized regression weights of variables for Model 4.

Endogenous variables		*β*	*B*	SE	*p*-values
Burnout (BurnoutScore)	Negative coping strategy (NCS)	0.238	0.020	0.006	<0.001
	Empathy (TEQScore)	−0.157	−0.121	0.063	0.056
Psychological distress (PDScore)	Negative coping strategy (NCS)	548	0.094	0.012	< 0.001
	Empathy (TEQScore)	−0.207	−0.331	0.155	0.033
Negative coping strategy (NCS)	Empathy (TEQScore)	0.088	0.819	0.977	0.402

*β = standardized regression weights; B = unstandardized regression weights; SE = standard error. Estimates for PDScore → BurnoutScore, PolyScore → PDScore, PolyScore → BurnoutScore remain the same as in Table 9, hence not included here.*

**Table 13:** The unstandardized and standardized estimates of direct, indirect, and total effects for variables relationships for Model 4.

Parameter	Variables relationship		Total (95% CI)	Direct (95% CI)	Indirect (95% CI)
Unstandardized	PolyScore	PDScore	−0.025 (−0.075, 0.031)^***^	−0.025 (−0.075, 0.031)^***^	—
	PolyScore	NCS	−0.008 (−0.016, −0.002)^*^	—	−0.008 (−0.016, −0.002)^*^
	PDScore		0.117 (0.094, 0.141)^**^	0.094 (0.069, 0.117)^**^	0.023 (0.009, 0.041)^**^
	Burnout Score		0.020 (0.007, 0.031)^**^	0.020 (0.007, 0.031)^**^	—
	Burnout Score	TEQScore	−0.105 (−0.252, 0.033)^***^	−0.121 (−0.263, 0.027)^***^	0.016 (−0.040, 0.068)^***^
	NCS		0.819 (−2.309, 2.710)^***^	0.819 (−2.309, 2.710)^***^	—
Standardized	PolyScore	PDScore	−0.059 (−0.179, 0.068)^***^	−0.059 (−0.179, 0.068)^***^	—
	PolyScore	NCS	−0.113 (−0.217, −0.022)^*^	—	−0.113 (−0.217, −0.022)^*^
	PDScore		0.683 (0.593, 0.772)^**^	0.548 (0.409, 0.661)^**^	0.135 (0.051.215)^**^
	Burnout Score		0.238 (0.085, 0.365)^**^	0.238 (0.085, 0.365)^**^	—
	Burnout Score	TEQScore	−0.136 (−0.322, 0.043)^***^	−0.157 (−0.342, 0.033)^***^	0.021 (−0.049, 0.086)^***^
	NCS		0.088 (−0.231, 0.295)^***^	0.088 (−0.231, 0.295)^***^	—

Bootstrap (1000) with 95% bias-corrected confidence interval: L = lower bound; U = upper bound; ^*^p-value <0.05; ^**^p-value <0.01; ^***^p-value >0.05. Estimates for POLY Score → Burnout Score, PDScore → Burnout Score, PolyScore → TEQScore, PDScore → TEQScore remain similar as in Table 10, hence not included here.

## Discussion

Our study demonstrates that professional behavior significantly decreases burnout levels among medical students, indicating that a strong understanding and practice of professionalism may mitigate the risk of burnout. The support for Hypothesis 1 suggests that higher levels of medical professionalism are associated with improved mental well-being, aligning with previous studies that have emphasized professionalism as a buffer against burnout in healthcare settings. Prior research showing a correlation between burnout and unprofessional behaviors, as well as compromised patient care standards (27–29). Specifically, our findings resonate with Brazeau et al. (30), who identified an inverse relationship between burnout levels and professionalism scores. Similarly, Dyrbye et al. (31) linked burnout with self-reported unprofessional behaviors among physicians. These results underscore the global prevalence of burnout in medical schools (32), and the inverse relationship we observed suggests that fostering a strong sense of professionalism in students may serve as a buffer against burnout, ultimately enhancing patient care standards. Our work highlights the critical role of professionalism in medical education, recommending that medical schools integrate comprehensive professionalism training to reduce burnout and improve healthcare outcomes.

Our study reveals that professional behavior demonstrates an indirect positive influence on the empathy levels of medical students by mitigating burnout. This finding aligns with global medical guidelines, such as those from the General Medical Council (GMC) in the UK and the Association of American Medical Colleges (AAMC), which emphasize the integral role of empathy in medical professionalism. Similar to our findings, previous studies have highlighted the relationship between reduced burnout and enhanced empathy among healthcare professionals. We postulate that professional behavior fosters a supportive learning environment, reducing stress and burnout, which in turn enhances empathy among medical students. Supporting this assumption, studies such as those by Shanafelt et al. (27) and Dyrbye et al. (33) have demonstrated that lower burnout levels correlate with higher empathy and better patient care. However, our study’s limitations include potential biases from self-reported measures and its cross-sectional design, which limits causal inferences. Despite these limitations, our findings highlight the importance of integrating professionalism training in medical curricula to foster empathy, reduce burnout, and ultimately improve patient care. We recommend that medical schools adopt comprehensive professionalism programs, which could have significant implications on fostering empathy, reducing burnout, and also improving medical education, thus leading to better patient outcomes and enhanced healthcare delivery.

Our study indicates that positive professional behavior indirectly reduces negative coping strategies (NCSs) among medical students. This finding aligns with existing literature suggesting that coping strategies are influenced by personality traits and attributional styles (34). Similar to our findings, previous research has shown that individuals with proactive, problem-oriented strategies tend to employ positive coping strategies (PCSs), while NCSs are negatively correlated with traits such as agreeableness and conscientiousness (35). We postulate that nurturing medical professionalism helps students avoid negative coping methods by promoting positive behaviors and encouraging social support, thereby enhancing their mental well-being. Supporting this assumption, the literature indicates that professionalism can foster a helpful educational atmosphere that mitigates stress and promotes healthy coping mechanisms (36). Our findings indicate the importance of incorporating professionalism training into medical curricula to promote healthy coping strategies. This has significant implications for medical education, suggesting that such training could advance learners’ mental health and overall well-being, ultimately leading to improved patient care.

This study highlights the significant impact of burnout on the coping strategies of medical students, influencing both positive coping strategies (PCSs) and negative coping strategies (NCSs). Similar findings have been reported globally, reflecting the increasing prevalence of emotional exhaustion among healthcare workers. For instance, studies in Saudi Arabia (37) revealed high rates of burnout and depression among residents, while research in Romania (38) found a significant burnout rate among medical students, particularly during the pandemic. Our findings align with global observations that burnout tends to influence coping strategies, with positive coping methods being more frequently utilized than negative ones in response to burnout, as observed in Singapore (39), the USA (40), Qatar (41), and Saudi Arabia (42, 43). We postulate that maintaining a positive attitude in the face of adversity is crucial for medical students to mitigate adverse effects on their mental health and enhance their overall performance. Supporting this assumption, evidence from various studies suggests that promoting positive coping strategies among students can contribute significantly to their emotional well-being and academic success (44). The findings indicate the importance of fostering positive coping mechanisms within medical education to support students’ mental health and academic performance. We recommend that medical schools integrate programs that promote positive coping strategies, which could have significant implications for improving the overall well-being and effectiveness of future healthcare professionals.

Our study reveals that psychological distress increases the use of positive coping strategies (PCSs) among medical students, which does. This finding resonates with existing literature that underscores the pivotal role of social support in safeguarding mental health across diverse populations, including medical students. Similar studies have highlighted the importance of social support in mitigating psychological distress and promoting positive coping mechanisms. For instance, research indicates that social support networks are crucial for medical students to manage stress effectively, as demonstrated in studies across various countries like China (45), Italy (46), Canada (47) and Australia (48). Conversely, inadequate support from family and friends has been linked to a higher risk of depression among medical students in the United States (49). We postulate that psychological distress may prompt medical students to actively seek out positive coping strategies, leveraging social support to enhance their mental resilience. This assumption is supported by literature emphasizing the beneficial effects of social support in reducing stress and improving coping mechanisms. Our findings highlight the critical role of fostering robust social support systems within medical education. We recommend that medical schools implement programs that strengthen social support networks and teach effective positive coping strategies, which could significantly enhance the mental well-being and academic success of medical students.

Our study highlights a substantial association between employing positive coping strategies (PCSs) and increasing the empathy levels of medical students, suggesting a positive correlation between coping and empathy. This finding is corroborated by previous research, such as that by Noda, Noda et al. (50), which also identified a link between coping mechanisms and empathy. Empathy, closely tied to coping and stress, inherently influences coping mechanisms that involve supportive activities or interactions with others (51). Furthermore, findings by Saha et al. (52) confirm a positive correlation between empathic self-efficacy and adaptive coping techniques, while noting a negative correlation with maladaptive coping strategies. We postulate that more empathetic medical students are inclined towards using positive coping strategies when faced with stressful situations or conflicts, as empathy promotes supportive and adaptive responses (51, 53). Supporting this assumption, the literature emphasizes the role of empathy in fostering positive coping behaviors. Our findings highlight the importance of fostering empathy among medical students to enhance their coping skills and overall well-being. We recommend that medical education programs integrate empathy training to encourage the use of positive coping strategies, which could significantly improve students’ mental health and professional performance.

Our study reveals a significant relationship between mental well-being issues and the adoption of negative coping strategies (NCSs) among medical students, particularly in response to burnout and psychological distress. This supports H4, and is consistent with earlier studies indicating that high levels of psychological distress, including depression, anxiety, and stress, positively correlate with the use of escape avoidance behaviors (54). Additionally, studies by Grynberg and López-Pérez (55) have similarly highlighted distress’s positive association with less healthy coping methods. We postulate that medical students experiencing mental well-being issues may resort to negative coping strategies as a means of escaping or avoiding stressful situations. This assumption is supported by literature emphasizing the detrimental impact of psychological distress on coping mechanisms, leading individuals to adopt maladaptive strategies in an attempt to cope with their emotions. Our findings highlight the importance of addressing mental well-being issues among medical students to mitigate the adoption of negative coping strategies. We recommend introducing extensive psychological and mental health support initiatives in medical education institutions to promote positive coping mechanisms and enhance overall student well-being. Such initiatives could have significant implications for improving the mental health and resilience of future health professionals, ultimately benefiting both students and the patients they serve.

Our study highlights a significant decrease in the empathy levels of medical students due to the direct and mediated effects of psychological distress. This finding aligns with previous research indicating that stress in medical practice can diminish empathy and overall performance quality (56). Similarly, Bellini et al. (57) found a negative correlation between distress, depression, and empathic concern. We postulate that anxiety, a component of psychological distress, may influence empathy due to heightened emotional sensitivity (58). Understanding this complex relationship between psychological functioning and empathy is crucial for medical students, as empathy fosters compassion and supportive care. Despite variations in research findings, empathy remains integral to positive social interactions and effective patient care. Our findings highlight the importance of addressing psychological distress among medical students to preserve empathy levels and enhance overall patient care. We recommend implementing interventions that promote mental well-being and resilience within medical education to safeguard empathy and improve health outcomes. Such initiatives could have significant implications for both medical students and the patients they serve, ultimately contributing to a more compassionate and effective healthcare system.

This study collectively presents some limitations that necessitate attention. Firstly, the reliance on self-reported data introduces potential biases, as participants’ experiences and behaviors may be overreported or overlooked due to social desirability or recall bias. Secondly, the cross-sectional design of these studies limits the ability to draw causal inferences between variables, as we cannot definitively establish the directionality of the relationships observed. Additionally, the diverse cultural and educational contexts in which the work was conducted may negatively influence the generalizability of the findings. The work also did not account for all potentially confounding variables that could impact the relationships between psychological distress, coping strategies, empathy, and professional behavior. Authors feel there is need for further longitudinal and multi-site research to confirm and expand upon these results.

This study presents several notable strengths that enhance their contribution to understanding the mental health and professional behavior of medical students. Firstly, they address a critical and timely issue by exploring the multifaceted impact of psychological distress, burnout, and coping strategies on empathy and professionalism in medical education. The use of established and validated measures for assessing psychological distress, coping strategies, empathy, and professional behavior ensures the reliability and validity of the data collected. Additionally, the integration of both direct and mediated effects in the analyses provides a comprehensive understanding of the complex relationships among these variables. The findings are consistently aligned with existing literature, further validating the results and highlighting the global relevance of promoting mental well-being and positive coping mechanisms in medical education. These insights underscore the importance of fostering a supportive environment for medical students, which has significant implications for improving their mental health, empathy, and overall professional performance.

Based on the findings, several key recommendations and implications emerge for enhancing the mental well-being and professional development of medical students. It is crucial to integrate wide-ranging mental health backing plans within medical education to address psychological distress, thereby reducing the adoption of negative coping strategies and preserving empathy levels. The promotion of professional behavior should be emphasized as it indirectly mitigates burnout and enhances empathy and positive coping strategies. Medical curricula should incorporate training that fosters empathy, resilience, and positive coping mechanisms, which are vital for managing stress and improving patient care. Additionally, fostering robust social support networks among medical students can significantly enhance their coping skills and overall well-being. These interventions not only improve the mental health and professional behavior of medical students but also have far-reaching implications for patient care and the healthcare system. By implementing these recommendations, medical schools can better prepare students to face the challenges of their profession, leading to more compassionate and effective healthcare professionals.

## Conclusion

Mental wellness issues significantly impact medical students’ health, learning, and patient care abilities. Stress, anxiety, depression, and burnout harm empathy and professionalism. While students often use PCSs, NCSs are also not uncommon and exacerbate challenges. Recognizing and addressing these difficulties with tailored support and training is crucial. Ensuring students’ quality of life and teaching effective coping mechanisms are essential for their future roles in patient care and societal contributions.

## Data Availability

The original contributions presented in the study are included in the article/Supplementary material, further inquiries can be directed to the corresponding author.
