# Supplementary material for: Decoding the interplay of medical professionalism, mental well-being, and coping in undergraduate medical students across culture: using structural equation modeling
Source: Front Med (Lausanne). 2024 Nov 5;11:1468654. doi: 10.3389/fmed.2024.1468654 (PMC11573547; doi:10.3389/fmed.2024.1468654)
Supplement: Supplementary file 1 [file Data_Sheet_1.docx]

**APPENDICES**

Appendix A (Part-1): Demographic for surveys

| **Tick (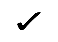) in the appropriate box:**  **Gender:** Male_____ Female____ Prefer not to say____  **Ethnicity:** Malay____ Chinese____ Indian____ Other____  **Year of Study**  **for Medical Course** 1^st^____ 3^rd^___ 5^th^____ |
| --- |

Appendix A (Part-2): Dundee Polyprofessionalism Inventory I: Academic Integrity

| 1 | Getting or giving help for course work against a teacher’s rule (e.g., Lending work to another student to look at) | |
| --- | --- | --- |
| 2 | Signing attendance sheets for absent friends or asking classmates to sign attendance sheets for you in labs or lectures | |
| 3 | Engaging in substance misuse (e.g., Drugs) | |
| 4 | Completing work for another student | |
| 5 | Lack of punctuality for classes | |
| 6 | Not doing the part assigned in group work. | |
| 7 | Examining patients without the knowledge or consent of supervising clinician | |
| 8 | Cheating in an exam by, e.g., copying from a neighbor, taking in crib material or using a mobile phone or getting someone else to sit for you | |
| **Dear student, if YOU or YOUR FRIENDS have ever done an act as mentioned in the above statement, which one of the following sanctions (1-10) you would like to recommend?** | | |
| **Rating scale** | | |
| 1. Ignore (None) | | 6. Failure of specific class/remedial work to gain credit |
| 2. Reprimand (verbal warning) | | 7. Failure of specific year (repetition allowed) |
| 3. Reprimand (written warning) | | 8. Expulsion from college (readmission after one year possible) |
| 4. Reprimand, plus mandatory counselling | | 9. Expulsion from college (no chance for readmission) |
| 5. Reprimand, counselling, extra work assignment | | 10. Report to a regulatory body |

Appendix A (Part-3): Depression, Anxiety, Stress Scale (DASS-9) (Yusoff, 2013a)

| 1 | (d) I found it difficult to work up the initiative to do things | 0 | 1 | 2 | 3 |
| --- | --- | --- | --- | --- | --- |
| 2 | (s) I tended to over-react to situations |  |  |  |  |
| 3 | (a) I experienced trembling (e.g., in the hands) |  |  |  |  |
| 4 | (a) I was worried about situations in which I might panic and make a fool of myself |  |  |  |  |
| 5 | (d) I felt that I had nothing to look forward to |  |  |  |  |
| 6 | (s) I found myself getting agitated |  |  |  |  |
| 7 | (s) I was intolerant of anything that kept me from getting on with what I was doing |  |  |  |  |
| 8 | (a) I felt I was close to panic |  |  |  |  |
| 9 | (d) I was unable to become enthusiastic about anything |  |  |  |  |
| **Rating scale DASS-9**  0=Did not apply to me at all  1=Applied to me to some degree, or some of the time  2=Applied to me to a considerable degree or a good part of time  3=Applied to me very much or most of the time  (d) = Depression  (s) = Stress  (a) = Anxiety | | | | | |
| DASS-9 simulated to DASS-42 scores.  The constants 3.6, 5.8, and 5.3 from below table were used to simulate the Depression, Anxiety, and stress scores from DASS-9 to simulate to get DASS-42 scores.  The total score of the DASS-9 approximately has to be multiplied by 5 respectively to simulate the original DASS-42 (Yusoff, 2013b).  m = multiplied by a constant to simulate the score of DASS-42. | | | | | |

|  | **Median** | **Mean (m)** | **SD** | **Range** |
| --- | --- | --- | --- | --- |
| **DASS-9** | | | | |
| **Depression** | **0** | **0.74 (3.6)** | **1.06** | **0 - 6** |
| **Anxiety** | **2** | **1.72 (5.8)** | **1.50** | **0 - 8** |
| **Stress** | **1** | **1.52 (5.3)** | **1.49** | **0 - 9** |
| **Total scale** | **3** | **3.98 (5.2)** | **3.18** | **0 - 18** |
|  |  |  |  |  |

The Following standard table for DASS-42 was used to categorize severity

**
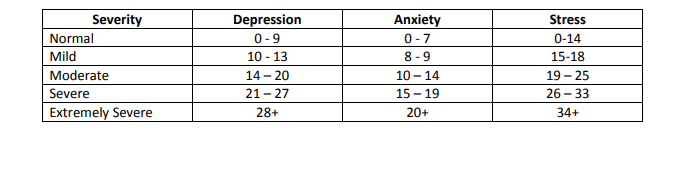
**

Appendix A (Part-4): Coping Orientation to Problems Experienced (Brief-cope)

| **Brief-COPE** | **Items** | | **I haven't been doing this at all** | **A Little bit** | **A medium amount** | **I’ve been doing this a lot** |
| --- | --- | --- | --- | --- | --- | --- |
| 1 | I've been saying to myself "this isn't real". 3 A | |  |  |  |  |
| 2 | I've been refusing to believe that it has happened. 8 A | |  |  |  |  |
| 3 | I've been using alcohol or other drugs to make myself feel better. 4 A | |  |  |  |  |
| 4 | I've been using alcohol or other drugs to help me get through it. 11A | |  |  |  |  |
| 5 | I've been getting emotional support from others. 5 E | |  |  |  |  |
| 6 | I've been getting comfort and understanding from someone .15 E | |  |  |  |  |
| 7 | I’ve been criticizing myself. 13 E | |  |  |  |  |
| 8 | I’ve been blaming myself for things that happened.26 E | |  |  |  |  |
| 9 | I've been trying to find comfort in my religion or spiritual beliefs. 22E | |  |  |  |  |
| 10 | I've been praying or meditating. 27E | |  |  |  |  |
| 11 | I've been learning to live with it. 24E | |  |  |  |  |
| 12 | I've been accepting the reality of the fact that it has happened. 20E | |  |  |  |  |
| Brief-COPE (Negative Coping items) | | 1, 2, 3, 4, 7 & 8 | | | | |
| Brief-COPE (Positive Coping items) | | 5, 6, 9, 10, 11 &12 | | | | |
| Scores are presented for three overarching coping styles as average scores (sum of item scores divided by number of items), indicating the degree to which the respondent has been engaging in that coping style.  = I haven’t been doing this at all  = A little bit  = A medium amount  = I’ve been doing this a lot  A normative percentile is presented based on data from a non-clinical sample of athletes (Poulus, Coulter, Trotter, & Polman, 2020). Interpretation by way of normative percentile helps contextualize results in comparison to typical responses of regular individuals.  In addition, a clinical percentile is presented which compares responses to clients receiving outpatient mental health services (Buchanan, 2021)  A percentile of 50, for example, represents an average score for a client in psychological therapy, whereas a percentile of 90 indicates that the respondents scored higher than 90 percent of other individuals in treatment. | | | | | | |

Appendix A (Part-5): Copenhagen Burnout Inventory (CBI)

|  | **CBI** | | | | | |
| --- | --- | --- | --- | --- | --- | --- |
|  | **Survey items** | **Response category and scoring** | | | | |
|  |  | Always^a^ or To a very high degree^b^  (Scoring 100)% | Often^a^ or To a high degree^b^  (Scoring 75)% | Sometimes^a^ or somewhat^b^ (Scoring 50)% | Seldom^a^ or To a low degree^b^  (Scoring 25)% | Never/almost never^a^ or To a very low degree^b^  (Scoring 0)% |
|  | ***Personal burnout*** |  |  |  |  |  |
| 1 | How often do you feel tired?^a^ |  |  |  |  |  |
| 2 | How often are you physically exhausted?^a^ |  |  |  |  |  |
| 3 | How often are you emotionally exhausted?^a^ |  |  |  |  |  |
| 4 | How often do you think: ”I can't take it anymore”?^a^ |  |  |  |  |  |
| 5 | How often do you feel worn out?^a^ |  |  |  |  |  |
| 6 | How often do you feel weak and susceptible to illness?^a^ |  |  |  |  |  |
|  | **Total average score** |  |  |  |  |  |
|  | ***Work-related burnout*** |  |  |  |  |  |
| 1 | Do you feel worn out at the end of the working day?^a^ |  |  |  |  |  |
| 2 | Are you exhausted in the morning at the thought of another day at work?^a^ |  |  |  |  |  |
| 3 | Do you feel that every working hour is tiring for you?^a^ |  |  |  |  |  |
| 4 | Do you have enough energy for family and friends during leisure time?^a^ (inverse scoring) |  |  |  |  |  |
| 5 | Is your work emotionally exhausting?^b^ |  |  |  |  |  |
| 6 | Does your work frustrate you?^b^ |  |  |  |  |  |
| 7 | Do you feel burnt out because of your work?^b^ |  |  |  |  |  |
|  |  |  |  |  |  |  |
| **Response Options:** Always - 100%, Often - 75%, Sometimes - 50%, Seldom - 25%, Never/almost never - 0% | | | | | | |
| **Total average score**  Possible score range for all scales is 0–100. a. Response categories for items denoted with^a^. b. Response categories for items denoted with^b^.  **Scoring Procedures**  The possible score range for all scales is 0 to 100. Scores for each subscale are averaged, and a total average score is calculated. | | | | | | |

Appendix A (Part-6): Toronto Empathy Questionnaire (TEQ)

| Please read each statement below carefully and rate how frequently you feel or act in the manner described. Circle your answer on the response form. There are no right or wrong answers or trick questions please answer each question as honestly as you can | | **Never** | **Rarely** | **Sometimes** | **Often** | **Always** |
| --- | --- | --- | --- | --- | --- | --- |
| 1 | When someone else is feeling excited, I tend to get excited too | 0 | 1 | 2 | 3 | 4 |
| 2 | Other people's misfortunes do not disturb me a great deal | 0 | 1 | 2 | 3 | 4 |
| 3 | It upsets me to see someone being treated disrespectfully | 0 | 1 | 2 | 3 | 4 |
| 4 | I remain unaffected when someone close to me is happy | 0 | 1 | 2 | 3 | 4 |
| 5 | I enjoy making other people feel better | 0 | 1 | 2 | 3 | 4 |
| 6 | I have tender, concerned feelings for people less fortunate than me | 0 | 1 | 2 | 3 | 4 |
| 7 | When a friend starts to talk about his\her problems, I try to steer the conversation towards something else | 0 | 1 | 2 | 3 | 4 |
| 8 | I can tell when others are sad even when they do not say anything | 0 | 1 | 2 | 3 | 4 |
| 9 | I find that I am "in tune" with other people's moods | 0 | 1 | 2 | 3 | 4 |
| 10 | I do not feel sympathy for people who cause their own serious illnesses | 0 | 1 | 2 | 3 | 4 |
| 11 | I become irritated when someone cries | 0 | 1 | 2 | 3 | 4 |
| 12 | I am not really interested in how other people feel | 0 | 1 | 2 | 3 | 4 |
| 13 | I get a strong urge to help when I see someone who is upset | 0 | 1 | 2 | 3 | 4 |
| 14 | When I see someone treated unfairly, I do not feel very much pity for them | 0 | 1 | 2 | 3 | 4 |
| 15 | I find it silly for people to cry out of happiness | 0 | 1 | 2 | 3 | 4 |
| 16 | When I see someone being taken advantage of, I feel kind of protective  towards him\her | 0 | 1 | 2 | 3 | 4 |
| Add each item results and divide total by 4 to find determine your score:  Higher scores indicate high levels of self-reported empathy while scores below 45 are indicative of below average empathy levels.  Scoring: positively worded Items (1, 3, 5, 6, 8, 9, 13, 16) are scored according to: Never = 0; Rarely = 1; Sometimes = 2; Often = 3; Always = 4. The negatively worded items (2, 4, 7, 10, 11, 12, 14, 15) are reverse scored. | | | | | | |

APPENDIX B: Measurement Model for Professional Behavior


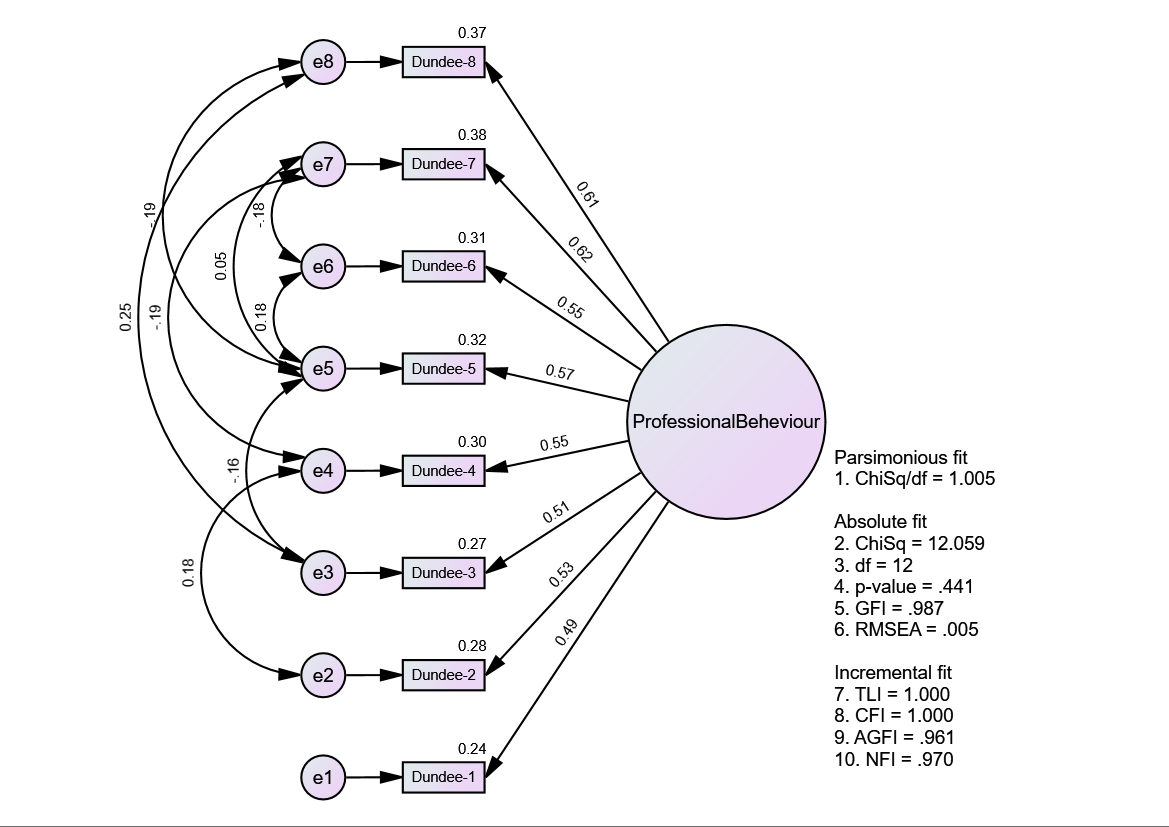


APPENDIX C: Measurement Model for MWB


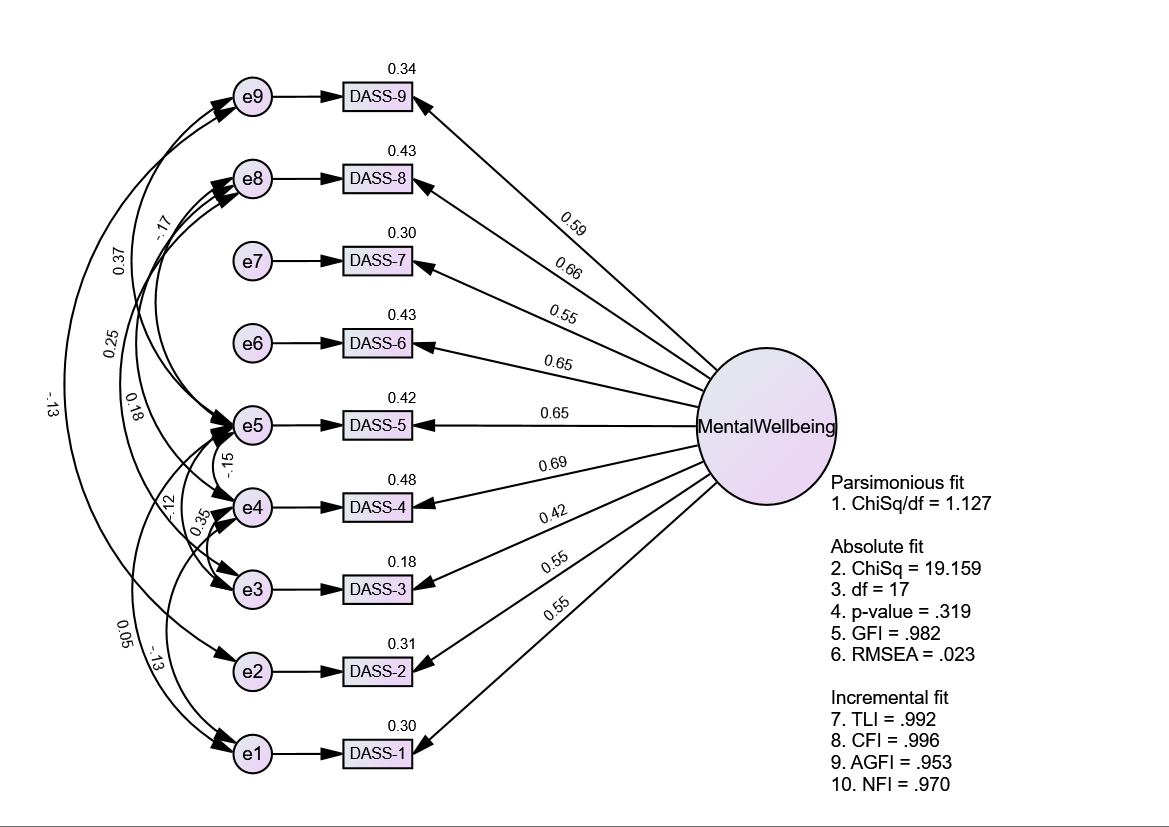


APPENDIX D: Measurement Model for PCS


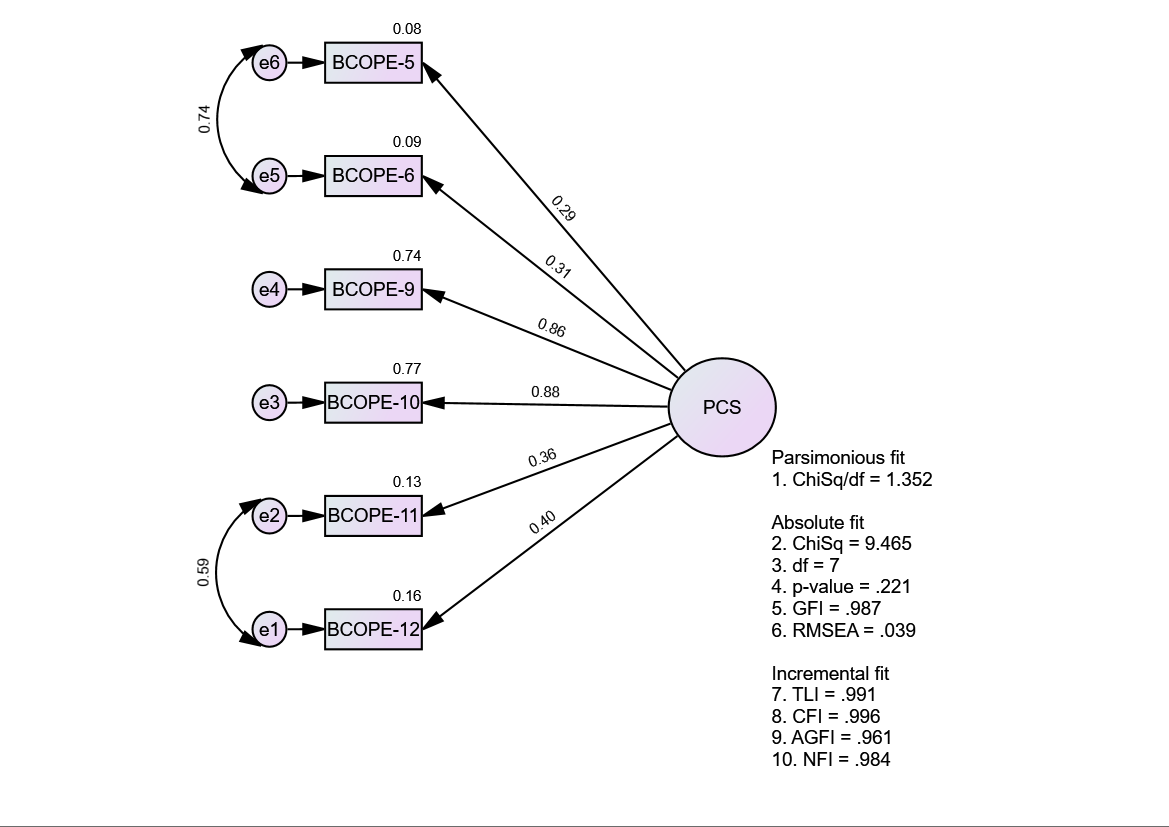


APPENDIX E: Measurement Model for NCS1


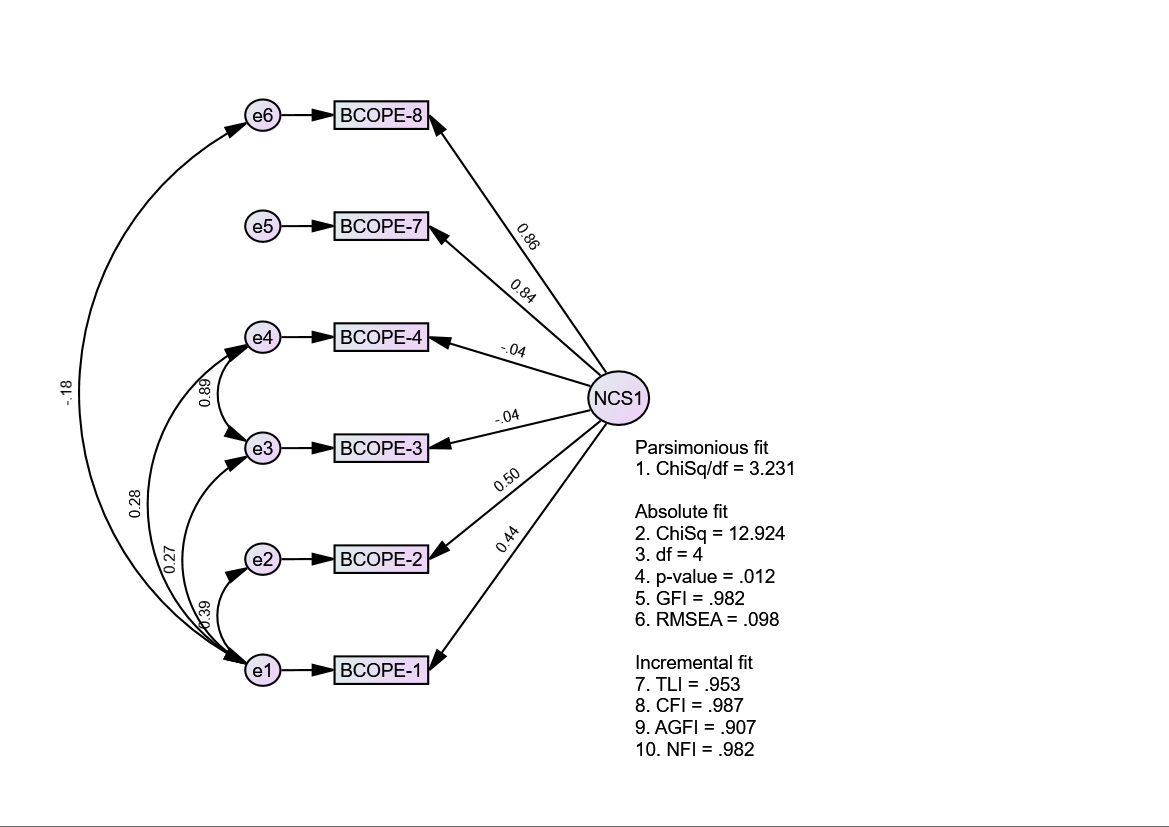


APPENDIX F: Measurement Model for NCS2


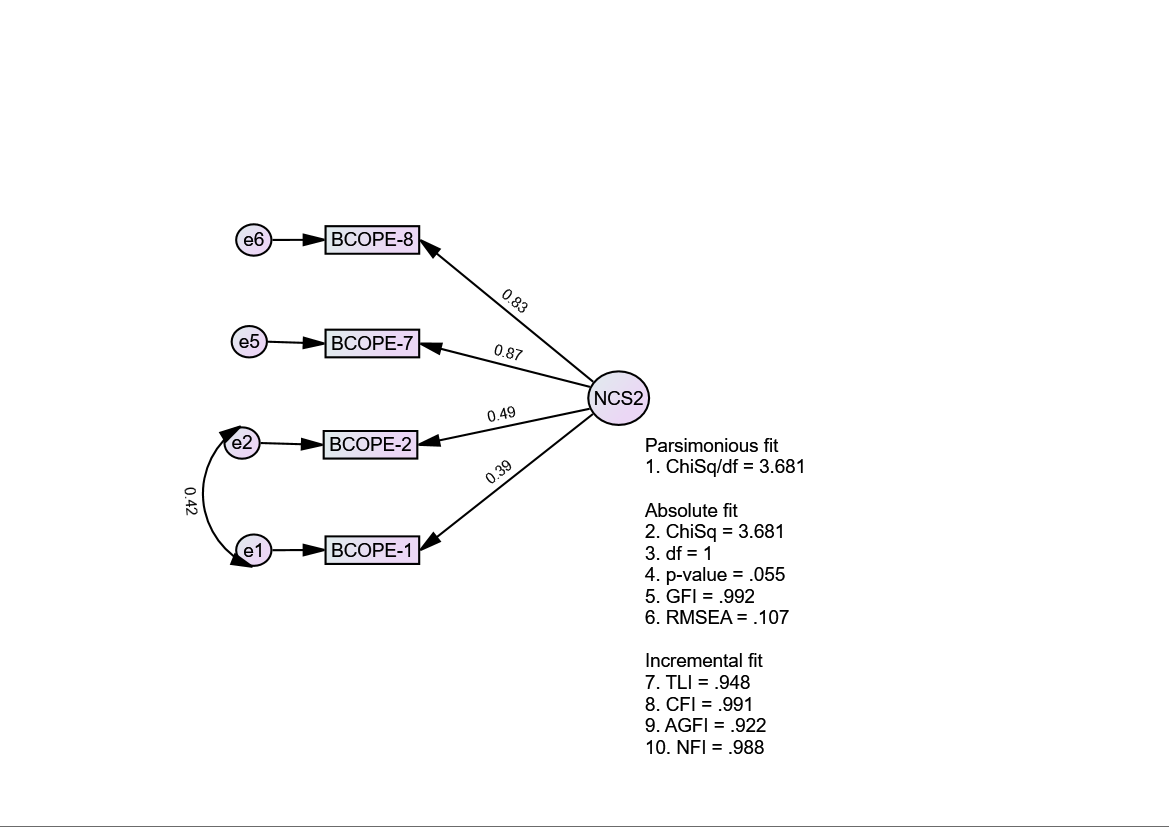


APPENDIX G: Measurement Model for Burnout


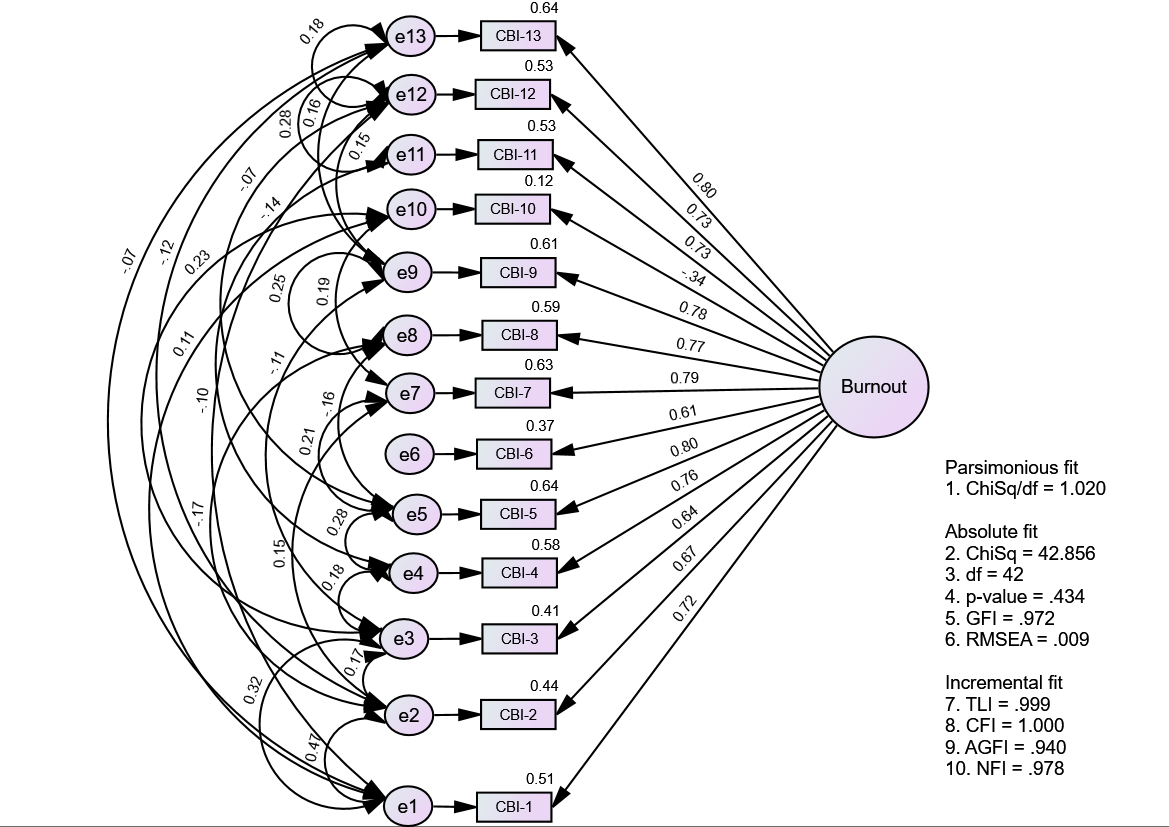


APPENDIX H: Measurement Model for Empathy


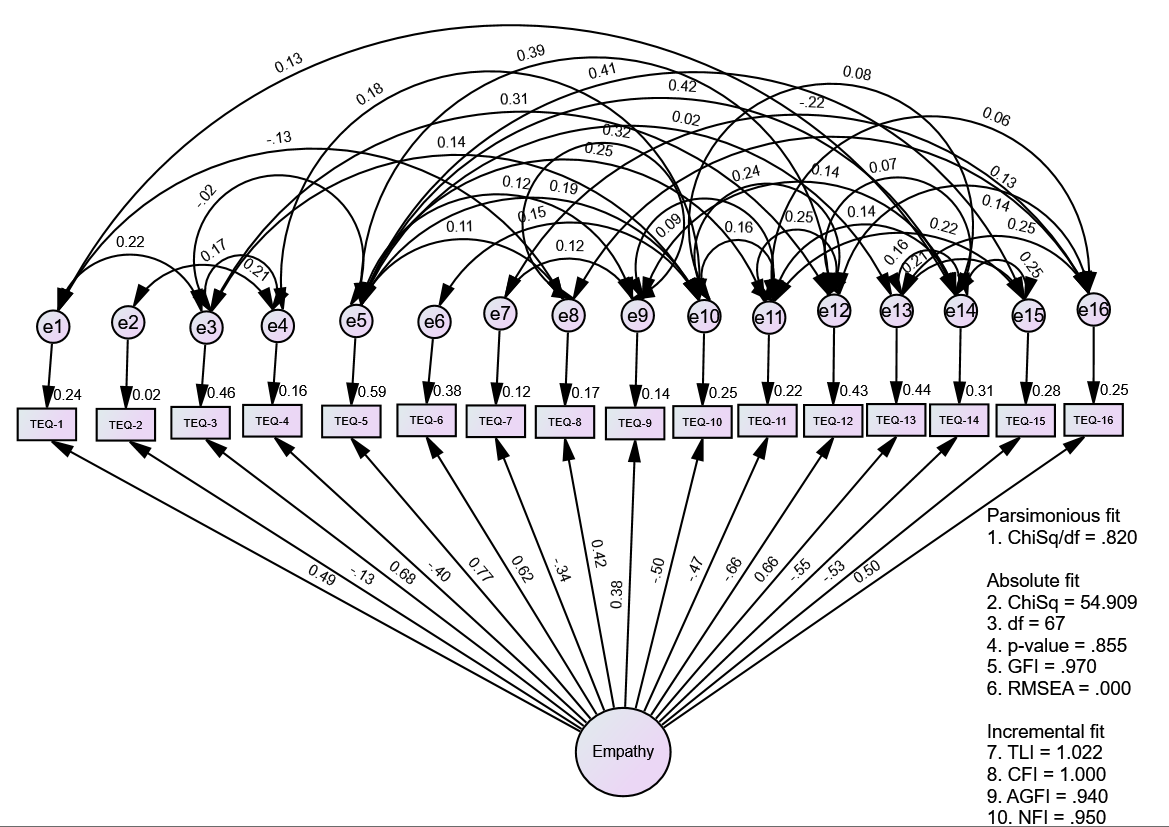


Appendix I

| Manifest  constructs | Latent  constructs | λ |
| --- | --- | --- |
| Dundee-1: Getting or giving help for coursework against a teacher’s rule (e.g., Lending work to another student to look at). | **Professional Behavior** | 0.49 |
| Dundee-2: Signing attendance sheets for absent friends or asking classmates to sign attendance sheets for you in labs or lectures. |  | 0.53 |
| Dundee-3: Engaging in substance misuse (e.g., Drugs). |  | 0.51 |
| Dundee-4: Completing work for another student. |  | 0.55 |
| Dundee-5: Lack of punctuality for classes. |  | 0.57 |
| Dundee-6: Not doing the part assigned in group work. |  | 0.55 |
| Dundee-7: Examining patients without the knowledge or consent of the supervising clinician. |  | 0.62 |
| Dundee-8: Cheating in an exam by, e.g., copying from a neighbor, taking in crib material using a mobile phone, or getting someone else to sit for you. |  | 0.61 |
| DASS-1: I found it difficult to work up the initiative to do things. | **Mental Well-being** | **0.55** |
| DASS-2: I tended to over-react to situations. |  | 0.55 |
| DASS-3: I experienced trembling (e.g., in my hands). |  | 0.42 |
| DASS-4: I was worried about situations in which I might panic and make a fool of myself. |  | 0.69 |
| DASS-5: I felt that I had nothing to look forward to. |  | 0.65 |
| DASS-6: I found myself getting agitated. |  | 0.65 |
| DASS-7: I was intolerant of anything that kept me from getting on with what I was doing. |  | 0.55 |
| DASS-8: I felt I was close to panic. |  | 0.66 |
| DASS-9: I was unable to become enthusiastic about anything. |  | 0.59 |
| NCS2-1: I've been saying to myself, "This isn't real. | **Coping** | 0.39 |
| NCS2-2: I've been refusing to believe that it has happened. |  | 0.49 |
| NCS2-7: I’ve been criticizing myself. |  | 0.87 |
| NCS2-8: I’ve been blaming myself for things that happened. |  | 0.83 |
| PCS-5: I've been getting emotional support from others. |  | 0.29 |
| PCS-6: I've been getting comfort and understanding from someone. |  | 0.31 |
| PCS-9: I've been trying to find comfort in my religion or spiritual beliefs. |  | 0.86 |
| PCS-10: I've been praying or meditating. |  | 0.88 |
| PCS-11: I've been learning to live with it. |  | 0.36 |
| PCS-12: I've been accepting the reality of the fact that it has happened. |  | 0.40 |

| CBI-1: How often do you feel tired? | Burnout | 0.72 |
| --- | --- | --- |
| CBI-2: How often are you physically exhausted? |  | 0.67 |
| CBI-3: How often are you emotionally exhausted? |  | 0.64 |
| CBI-4: How often do you think: “I can't take it anymore? |  | 0.76 |
| CBI-5: How often do you feel worn out? |  | 0.80 |
| CBI-6: How often do you feel weak and susceptible to illness? |  | 0.61 |
| CBI-7: Do you feel worn out at the end of the working day? |  | 0.79 |
| CBI-8: Are you exhausted in the morning at the thought of another day at work? |  | 0.77 |
| CBI-9: Do you feel that every working hour is tiring for you? |  | 0.78 |
| CBI-10: Do you have enough energy for family and friends during leisure time?” |  | -.34 |
| CBI-11: Is your work emotionally exhausting? |  | 0.73 |
| CBI-12: Does your work frustrate you? |  | 0.73 |
| CBI-13: Do you feel burnt out because of your work? |  | 0.80 |
| TEQ-1: When someone else is feeling excited, I tend to get excited too. | **Empathy** | 0.49 |
| TEQ-2: Other people's misfortunes do not disturb me a great deal. |  | -.13 |
| TEQ-3: It upsets me to see someone being treated disrespectfully. |  | 0.68 |
| TEQ-4: I remain unaffected when someone close to me is happy. |  | -.40 |
| TEQ-5: I enjoy making other people feel better. |  | 0.77 |
| TEQ-6: I have tender, concerned feelings for people less fortunate than me. |  | 0.62 |
| TEQ-7: When a friend starts to talk about his\her problems, I try to steer the conversation towards something else. |  | -.34 |
| TEQ-8: I can tell when others are sad, even when they do not say anything. |  | 0.42 |
| TEQ-9: I find that I am "in tune" with other people's moods. |  | 0.38 |
| TEQ-10: I do not feel sympathy for people who cause their own serious illnesses. |  | -.50 |
| TEQ-11: I become irritated when someone cries. |  | -.47 |
| TEQ-12: I am not really interested in how other people feel. |  | -.66 |
| TEQ-13: I get a strong urge to help when I see someone who is upset. |  | 0.66 |
| TEQ-14: When I see someone treated unfairly, I do not feel very much pity for them. |  | -.55 |
| TEQ-15: I find it silly for people to cry out of happiness. |  | -.53 |
| TEQ-16: When I see someone being taken advantage of, I feel kind of protective towards him\her. |  | 0.50 |

Note: λ = Standardized regression weights
